# Treatment of Cyanide-Free Wastewater from Brass Electrodeposition with EDTA by Electrodialysis: Evaluation of Underlimiting and Overlimiting Operations

**DOI:** 10.3390/membranes10040069

**Published:** 2020-04-11

**Authors:** Kayo Santana Barros, Tatiana Scarazzato, Valentín Pérez-Herranz, Denise Crocce Romano Espinosa

**Affiliations:** 1 Department of Chemical Engineering, University of São Paulo (USP), Av. Professor Lineu Prestes, 580, Bloco 18–Conjunto das Químicas, São Paulo–SP 05434-070, Brazil; espinosa@usp.br; 2 IEC Group, ISIRYM, Universitat Politècnica de València–Spain, Camí de Vera s/n, 46022, P.O. Box 22012, E-46071 València, Spain; vperez@iqn.upv.es; 3 Department of Materials Engineering, Federal University of Rio Grande do Sul (UFRGS), Av. Bento Gonçalves, Porto Alegre 91501-970, Brazil; tatiana.scarazzato@gmail.com

**Keywords:** electrodialysis, chronopotentiometry, ion-exchange membrane, overlimiting current, water dissociation

## Abstract

Growing environmental concerns have led to the development of cleaner processes, such as the substitution of cyanide in electroplating industries and changes in the treatment of wastewaters. Hence, we evaluated the treatment of cyanide-free wastewater from the brass electroplating industry with EDTA as a complexing agent by electrodialysis, aimed at recovering water and concentrated solutions for reuse. The electrodialysis tests were performed in underlimiting and overlimiting conditions. The results suggested that intense water dissociation occurred at the cathodic side of the commercial anion-exchange membrane (HDX) during the overlimiting test. Consequently, the pH reduction at this membrane may have led to the reaction of protons with complexes of EDTA-metals and insoluble species. This allowed the migration of free Cu^2+^ and Zn^2+^ to the cation-exchange membrane as a result of the intense electric field and electroconvection. These overlimiting phenomena accounted for the improvement of the percent extraction and percent concentration, since in the electrodialysis stack employed herein, the concentrate compartments of cationic and anionic species were connected to the same reservoir. Chronopotentiometric studies showed that electroconvective vortices minimized fouling/scaling at both membranes. The electrodialysis in the overlimiting condition seemed to be more advantageous due to water dissociation and electroconvection.

## 1. Introduction

Electrodeposited brass coatings are widely used for decorative purposes, the protection of steel and the promotion of rubber adhesion to steel and other metals [1]. For achieving the simultaneous electrodeposition of copper and zinc, complexing agents are used to reduce the activity of the noblest ion, Cu^2+^, bringing the reduction potentials of both metals closer together [2]. Conventionally, cyanide has been used in brass electrodeposition due to its ability to form very strong and stable complexes with metals in solution [3]. However, its high toxicity and the need for a rigorous maintenance and control of its solutions have prompted an effort to discover eco-friendly complexing agents able to produce brass deposits with similar quality.

Some alternative complexing agents already tested in brass electrodeposition are glycerol [4,5], glycine [1,3], sorbitol [6,7], ethylenediaminetetraacetic acid (EDTA) [8,9], citrate [10], pyrophosphate [11], pyrophosphate-oxalate [12], triethanolamine [2], glucoheptonate [13], nitrilotriacetic acid [14], tartrate [15], choline acetate [16], bis(trifluoromethylsulfonyl)imide [17] and d-mannitol [18]. Among them, EDTA is interesting since it is widely used as a complexing agent in the electrodeposition of metals [19,20] and for separating cations using electrodialysis, exploiting a difference in the solubility constants of the complexes [21,22].

The use of EDTA in the cyanide-free electrodeposition of copper-zinc on steel was evaluated by Almeida et al. [8,9]. The authors investigated the influence of the deposition potential and the bath composition (proportion of Cu/Zn) on the morphology and phase composition of the Cu-Zn deposits. Considering the promising use of electrodialysis for treating the wastewater generated in this electrodeposition, Barros et al. [23] recently employed chronopotentiometry to study the transport properties of the complexes present in the rinsing water generated in this brass electroplating, which is the main wastewater from the process. The authors assessed the influence of the solution pH, Cu^2+^/Zn^2+^ proportion and EDTA/Cu^2+^ molar ratio on the limiting current density (*i_lim_*), ohmic resistance, plateau length, concentration polarization and precipitate formation using the anion-exchange membrane HDX200. However, the treatment of the wastewater generated in brass electrodeposition with EDTA has not been studied yet.

Considering the limitations involved in the use of chemical precipitates for treating wastewaters from electroplating industry, there has been a growing interest in technologies that can enable water reclamation as well as the recovery and reuse of metal ions from electroplating wastewaters, such as electrodialysis [24,25]. 

**Figure 1:**
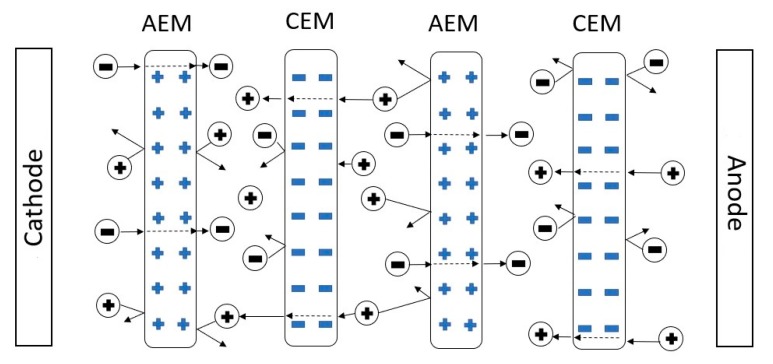
A typical electrodialysis system with cation exchange membranes (CEMs) and anion exchange membranes (AEMs).

Electrodialysis (ED) is an electrochemical process able to separate ions from a solution by an applied electric potential difference. Cation- and anion-exchange membranes (CEM and AEM, respectively) are arranged in an alternating pattern between the cathode and anode to form individual compartments, as shown in Figure 1. The positively charged cations migrate towards the cathode and negatively charged anions towards the anode. Considering an ideal system, CEMs only allow only the passage of cations, whereas AEMs only allow the passage of anions. Therefore, as the electrodialysis is performed, solutions more concentrated and diluted than the original are obtained. 

Traditionally, electrodialysis is conducted under underlimiting current regimes, under between 70% and 80% of the limiting current density of the membrane/electrolyte system to avoid concentration polarization phenomena [26]. Nevertheless, some authors have recently assessed the operation of ED at overlimiting current densities, since an improved ionic transport through the membranes has been observed [27,28]. Overlimiting current densities were verified to be responsible for an additional supply of counterions on the membrane surface, which occurs because of gravitational convection, electroconvection, water dissociation and the exaltation effect [29,30]. Electroconvection is one of the major phenomena responsible for ion transport when the system operates under an overlimiting current regime, since it allows a better availability of ions at the membrane interface by supplying “fresh” solution to the membrane surface and by removing the depleted solution [31,32]. Besides the improvement in ion transfer, the ED operation with intensive current may reduce the membrane area, which is considerably advantageous, since the costs of ion-exchange membranes are generally high. In turn, operating at overlimiting current densities may lead to intense water dissociation at the membrane surface, which favors the deposition of organic and inorganic substances on it (fouling and scaling, respectively) [33,34]. 

To guarantee the effectiveness of the electrodialysis, it is important to evaluate certain membrane properties by using dynamic characterization methods. Among the possibilities, chronopotentiometry is a valuable technique for investigating kinetic effects, such as transport phenomena and electrochemical reactions. This technique allows the determination of the limiting current density, electrical resistance and plateau length and the evaluation of the tendency of precipitates to form at the membrane interface [35]. Moreover, it is important to assess the influence of the membrane’s time of use in ED on its properties and on its ability to transport ions. 

Considering the advantages related to the use of electrodialysis for treating wastewaters and the promising substitution of cyanide by EDTA in brass electrodeposition, the present paper aims at employing ED to treat synthetic solutions of the wastewater from brass electrodeposition with EDTA as a complexing agent. Two electrodialysis tests were performed: one in an underlimiting and the other in an overlimiting condition. Prior to the tests, the limiting current density of the membranes/electrolyte system was determined by constructing current–voltage curves. The concentration of the working solution was based on the electrodeposition bath tested by Almeida et al. [8,9]. We here used a closed system configuration of electrodialysis for performing concentration tests, for obtaining a concentrated solution rich in cations and anions, and a diluted solution [36,37]. The ED configuration was chosen based on the possibility of replacing a portion of desalted solution with a portion of a new solution with the same initial content of components and the recovery of metals from the concentrated solution in the electrodeposition bath. To the best of our knowledge, there is no paper in the literature that evaluates this configuration in an overlimiting condition. The results were assessed in terms of percent extraction of the species from the synthetic wastewater, percent concentration—that is, the recovery of copper-EDTA and zinc-EDTA complexes—and a mass balance that was performed at different stages of the experiments. After the ED tests, chronopotentiometric studies were performed for evaluating the influence of the underlimiting and overlimiting regimes on the membrane properties, such as the limiting current density, electrical resistance, transition time and fraction of conductive area.

## 2. Materials and Methods

### 2.1. Electrodialysis Bench System

**Figure 2:**
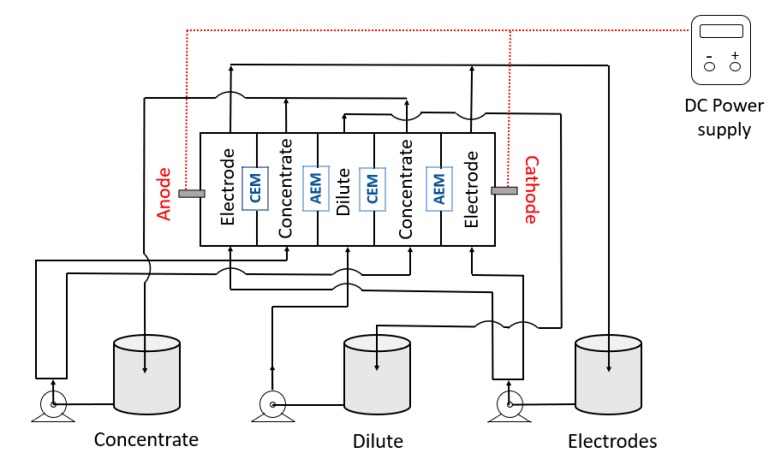
A schematic representation of the electrodialysis (ED) system used.

**Figure 3:**
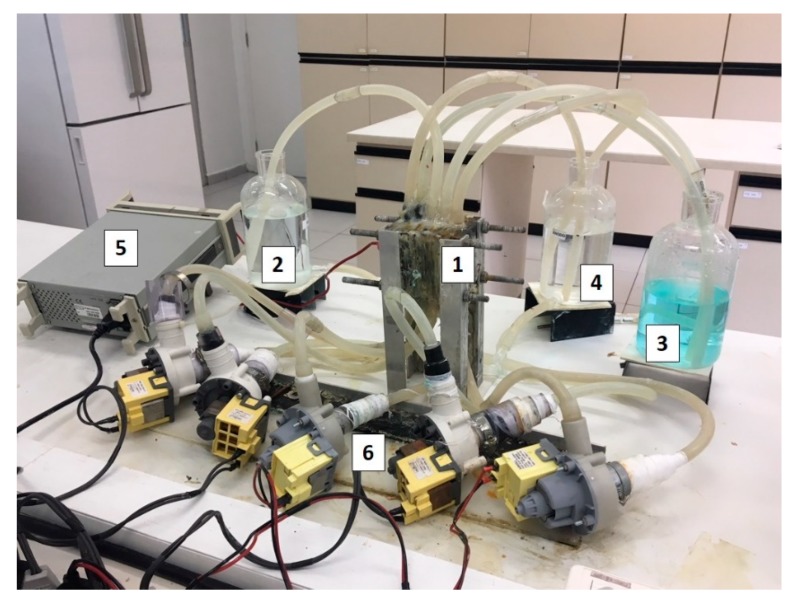
The electrodialysis system with five compartments: 1 is the ED cell; 2 is the synthetic rinsing water; 3 and 4 are the concentrated and electrode solutions, respectively; 5 is the DC power supply and 6 refers to the pumps.

The electrodialysis tests were carried out in a home-made five-compartment ED cell made of acrylic, separated by cation- and anion-exchange membranes with an active area of 16 cm^2^, arranged alternately in a “Cathode (electrode)-AEM-CEM-AEM-CEM-Anode (electrode)” configuration. The flow channel width between two membranes was 1 cm. The five compartments were 8 cm × 8 cm × 1 cm in dimension and were connected to three 1 L independent reservoirs. The reservoirs with the solution to be treated and with the solution to be concentrated were labeled as diluted and concentrated solutions, respectively, and both were fed with the synthetic wastewater (working solution). The reservoir connected to the electrode compartments was fed with a Na_2_SO_4_ solution to maintain the electrical conductivity. All the reservoirs were independently connected to centrifugal electro-pumps to produce the circulation of the solutions (80 L.h^−1^). A schematic representation of the electrodialysis system employed is presented in Figure 2, whereas the real ED system used is shown in Figure 3. The electrodes were made of titanium coated with titanium and ruthenium oxides (70RuO_2_/30TiO_2_, De Nora, Sorocaba, Brazil) and present an active area of 16 cm^2^ (4 cm × 4 cm). Both electrodes were placed at the extremities and connected to an external power source. The determination of the limiting current density of the membranes was performed as described in Section 2.3.

### 2.2. Experimental Procedure

Two electrodialysis experiments were performed: the underlimiting and overlimiting tests. Initially, both dilute and concentrate compartments were fed with the working solution of the synthetic rinsing water, and the electrodes compartment with the conductive solution of NaSO_4_. A current density value established based on the current–voltage curves of both membranes was applied for transporting ions from the dilute compartment to the concentrate one. In the underlimiting test, the applied current density was 80% of the limiting current density (*i_lim_*) of the cation-exchange membrane (CEM), which was determined for the initial working solution. In the overlimiting test, the current density was 120% of the *i_lim_* of the anion-exchange membrane (AEM). This procedure was carried out until the conductivity of the diluted solution reached values close to those for tap water (~0.2 mS/cm). When this condition was reached, the diluted solution was replaced with the working solution and the experiment was conducted again, until its conductivity also reached ~0.2 mS/cm. Each renewal of the diluted solution was named a “cycle”. Four cycles were performed due to the occurrence of ionic transfer limitation by the diffusion mechanism between the dilute and concentrate compartments. The conductivity and pH of the three solutions were monitored throughout the cycles. At the end of each cycle, the diluted solution and 10 mL samples of the concentrate compartment were collected and forwarded for chemical analyses. The ED performance was evaluated as a function of percent extraction (PE%) and percent concentration (PC%), according to Equation (1) and Equation (2), where $C_{0}^{j}$ and $C_{t}^{j}$ are the concentrations of an ion *j* in the initial state and at a given time, respectively. As PE% was calculated with data from the diluted solutions, the values obtained were lower than 100%. For PC%, the values obtained after the first cycle were higher than 100% since they were calculated with data from the concentrate compartment after each cycle, which means they were accumulative.

(1) $$PE\%=\left(1-\frac{C_{t}^{j}}{C_{0}^{j}}\right).100$$

(2) $$PC\%=\left(\frac{C_{t}^{j}}{C_{0}^{j}}-1\right).100$$

After the last cycle, the membranes were forwarded for chronopotentiometric tests, as described in Section 2.6.

### 2.3. Determination of the Limiting Current Density of the Membrane/Electrolyte System in the ED Stack

For defining the current to be applied to the electrodialysis tests, current–voltage curves (CVCs) for both membranes were constructed using the same apparatus of the home-made ED experiments. Platinum wires without an inert braid were placed at the interfaces of the anion- and cation-exchange membranes between the dilute and concentrate compartments. Voltmeters were connected directly to the platinum wires of both membranes. This configuration has already been used by other authors for determining the limiting current densities of membrane/electrolyte systems [26,38]. 

Two CVCs were constructed: one for the anion-exchange membrane and another for the cation-exchange membrane. The curves were obtained by increasing the applied current densities (*i*) gradually every 2 min, in 2 mA steps, with an interval of 3 min without the application of current. The potential drop (U_m_) between the AEM and CEM was measured immediately before the interruption of the current densities. Before the experiments, the membranes were equilibrated for 24 h in the solutions to be subsequently used. The experiments were performed in duplicate.

The limiting current densities of both membranes were determined by the intersections of the tangential lines of the first and second regions of the CVC, as described elsewhere [38]. 

### 2.4. Ion-Exchange Membranes

The commercial anion- and cation-exchange membranes used in the ED system were HDX200 and HDX100 (Hidrodex, Garça, Brazil), respectively. Both are heterogeneous. The HDX200 membrane contains quaternary amine groups attached to the membrane matrix, and the HDX100 membrane has sulfonic acid as fixed groups. The characteristics of both membranes are described elsewhere [38].

### 2.5. Working Solutions

The synthetic rinsing water evaluated herein was prepared by the dilution of the bath solution assessed by Almeida et al. [8,9] in their study of brass electrodeposition using EDTA as a complexing agent and a Cu^2+^/Zn^2+^ proportion of 30%.

The bath solution was prepared with CuSO_4_.5H_2_O (0.06 mol/L), ZnSO_4_.7H_2_O (0.14 mol/L), EDTA disodium salt (0.15 mol/L) and NaOH (3 mol/L) (Labsynth, Diadema, Brazil). The working solution that simulated the rinsing water was prepared by diluting the electrodeposition bath solution at a 1% v/v proportion in distilled water, and this solution was used to feed the dilute and concentrate reservoirs. Table 1 presents the initial conditions of the working solution used.

A Na_2_SO_4_ solution was used to feed the electrodes compartment. The conductivity of this solution was 11 mS/cm, about twice as great as the conductivity of the working solution, to reduce the resistance of the system. During the experiments, drops of NaOH solution (40% wt) were added to the electrode reservoir to minimize the influence of oxidation-reduction reactions on the solution conductivity and to maintain this parameter at approximately 11 mS/cm.

### 2.6. Chronopotentiometric Measurements

After the electrodialysis tests, the membranes were forwarded for chronopotentiometric steps. Firstly, they were immersed separately in the working solution and equilibrated for 24 h. Then, chronopotentiometric experiments were performed for evaluating the influence of each ED performed on the transport properties of the cation- and anion-exchange membranes. Herein, the virgin membranes not exposed to electrodialysis were also evaluated by chronopotentiometry.

The chronopotentiometric experiments were performed using a three-compartment cell with a cation- and anion-exchange membrane separating the central compartment from the cathode and anode, respectively. Two graphite electrodes were placed at the extremities of the cell, and during the experiments, some current values were imposed by a potentiostat/galvanostat (Autolab, PGSTAT 20, Utrecht, The Netherlands). Ag/AgCl reference electrodes (Sensoglass, São Paulo, Brazil) immersed in Luggin capillaries were installed on each side of the membrane for measuring the potential drop across the membranes. The experiments were conducted in duplicate, at room temperature and without stirring. For constructing the chronopotentiograms, current pulses were applied for 300 s. Then, the relaxation process was allowed to proceed for 100 s before the next pulse was applied. These durations were chosen based on previous work [37,39]. The current–voltage curves in the chronopotentiometric step were obtained from the steady-state polarization voltage of the membranes (potential drop) corresponding to each current pulse. A schematic representation of the chronopotentiometric system can be found elsewhere [39]. 

### 2.7. Analytical Methods

The concentration of copper and zinc ions was determined by energy dispersive X-ray fluorescence spectrometry (PANalytical Epsilon 3XL, Almelo, The Netherlands). The concentrations of sodium (Na^+^) and sulfate (SO_4_^2−^) were ascertained by ion chromatography (IC 858, Metrohm, Herisau, Switzerland). Finally, the concentration of EDTA was determined by Total Organic Carbon analysis (TOC-L, Shimadzu, Columbia, United States of America). Before the analyses, the solutions were filtered. Data represent the averages of three analyses performed, and the estimated relative error between the concentration values was below 5%.

During the experiments, the conductivity and the pH of the solutions in the three reservoirs were monitored with an electrical conductivity meter (Sensoglass, São Paulo, Brazil) and a PH21 pH meter (Hanna, Barueri, Brazil), respectively.

## 3. Results

**Figure 4:**
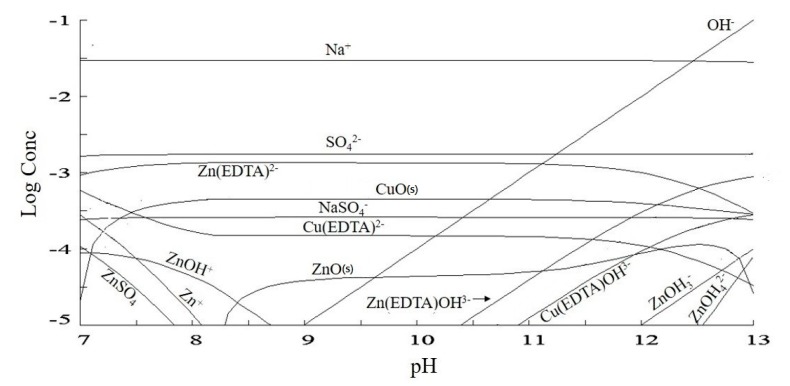
A speciation diagram constructed with the composition of the working solution.

For understanding the influence of the species present in solution on the properties of the membranes, a speciation diagram for the initial composition of the working solution was constructed with the aid of the Hydra-Medusa software [40] (Figure 4). Then, the concentrations of the main ionic species in the initial solution (under pH 12.25) were determined, and the results are presented in Table 2. As observed, the species present in the highest concentration is the cation Na^+^, which is the only species in solution that theoretically crosses the cation-exchange membrane. Regarding the anionic species, those present in the highest concentrations in the solution are OH^−^, SO_4_^2−^, Zn(EDTA)^2−^ and Zn(EDTA)OH^3−^. The presence of two insoluble species can also be observed: CuO and ZnO, which influenced the results for percent extraction and percent concentration.

### 3.1. Obtaining the Current–Voltage Curves in the Stack of Electrodialysis

**Figure 5:**
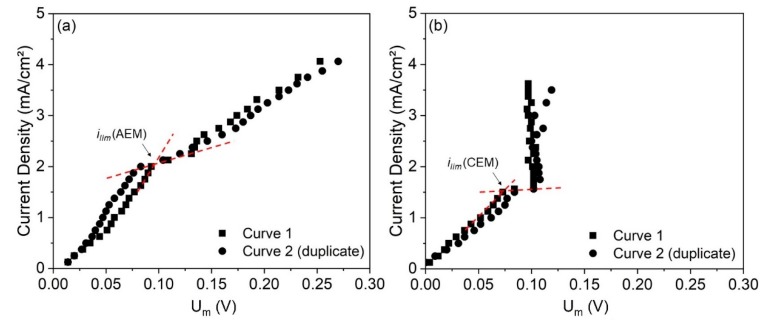
Current–voltage curves in duplicate of the (**a**) anion-exchange membrane (AEM) and (**b**) cation-exchange membrane (CEM).

The CVCs of the anion- and cation-exchange membranes were constructed in the electrodialysis stack for determining their limiting current density. The curves obtained are depicted in Figure 5 and the error in the *i_lim_* determination between the duplicate curves was 3.5% and 0.7% for the AEM and CEM, respectively. 

As observed, the AEM curve (Figure 5a) presented two limiting current densities: one at 0.5 mA/cm^2^ and the other at 2.0 mA/cm^2^. This behavior was already seen for ampholyte-containing solutions due to the different forms of species depending on the local pH [41] and for other systems where the prevailing species that passes through the membranes changes as the current density is increased [42,43]. The species that passes at each current density depends on its size, molar concentration and mobility/diffusion coefficient. Here, the first limiting current density for the AEM (*i_lim1,AEM_* = 0.5 mA/cm^2^) must be related to the depletion of OH^−^ ions, due to the greater concentration and mobility of these ions (Table 2), whereas the second one (*i_lim2,AEM_* = 2.0 mA/cm^2^) must be related to the depletion of anionic species present in lower concentrations, such as SO_4_^2−^ and complexes with EDTA. Then, a plateau was reached, and the third region showed the usual linear behavior. For defining the current applied to the electrodialysis test in the overlimiting condition, we considered the *i_lim_* of the AEM as 2.0 mA/cm^2^, since only at current densities above this can we see the third region of the current–voltage curve, where overlimiting phenomena occur.

For the cation-exchange membrane (Figure 5b), the curve showed a linear relationship in the first region, but in the third one, the potential drop remained practically constant with the increase in current density. This occurred due to the very fast and intense ionic transport through the CEM under overlimiting conditions, as will be discussed. Despite the absence of the linear behavior in the third region, it was possible to determine the limiting current density for the CEM (i*_lim,CEM_* = 1.5 mA/cm^2^), since the change in the slope after the first region was evident. 

Finally, the higher value of limiting current density for the AEM was due to the greater concentration of the anionic species than of the cationic species in the solution (Table 2), besides the high mobility of OH^−^.

### 3.2. Electrodialysis

Two electrodialysis experiments were performed. Table 3 presents the current density applied to each test performed, with the relationships between these current densities and the limiting current densities of both membranes. 

As observed, in the underlimiting experiment, the applied current density was lower than the *i_lim_* of both membranes (80% and 60% of the *i_lim_* of the CEM and AEM, respectively). In the overlimiting experiment, both membranes were in overlimiting conditions, since the *i* applied was 60% and 20% above the *i_lim_* of the CEM and AEM, respectively. The limiting current densities were determined with the working solution in its initial state. As the concentration of the dilute compartment decreased throughout the tests, the limiting current density also decreased. Hence, in the underlimiting test, the system may have operated in the overlimiting condition when the applied current density surpassed the limiting current density of the membranes/electrolyte system.

**Figure 6:**
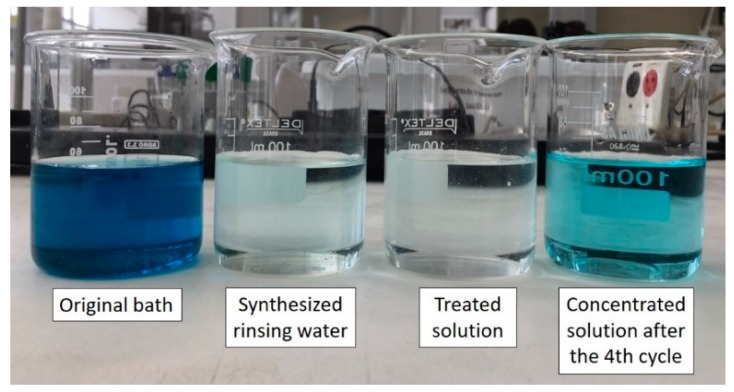
The visual aspects of the original bath solution, the synthetic rinsing water, the treated solution (diluted) and the concentrated solution after the fourth cycle of the overlimiting test.

Figure 6 presents the visual aspects of the four solutions involved in the overlimiting test: the original bath solution before its dilution, the synthetic rinsing water (or working solution), the diluted (treated) and concentrated solutions obtained after the fourth cycle.

#### 3.2.1. Evaluation of the Conductivity

**Figure 7:**
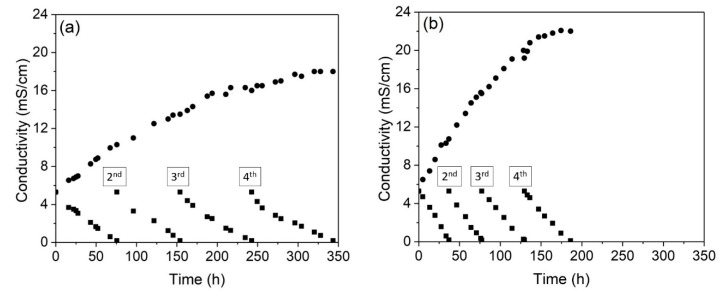
The conductivity of the concentrated (●) and diluted (■) solutions during the four cycles of the (**a**) underlimiting and (**b**) overlimiting tests.

Figure 7 presents the conductivity of the concentrate and dilute compartments throughout the four cycles performed in the ED tests. As expected, the increase in the current density strongly decreased the operation time, from 344 h to 186 h. In relation to the conductivity of the concentrate compartment after each cycle, Figure 7 shows that the overlimiting test accounted for the highest conductivity achieved in all cycles. As the conductivity of the dilute compartment was the same after all the cycles in the experiments (~0.2 mS/cm), the difference in the conductivity of the concentrated solutions suggests that the increase in the current density caused a change in the type of species that preferentially passed through the membranes. This is also going to be shown in the chemical analyses. The final pH values of the diluted and concentrated solutions after each cycle of the electrodialysis tests are presented in Table 4.

#### 3.2.2. Percent Concentration and Percent Extraction

Table 5 presents the concentration, in ppm, of the species copper, zinc, EDTA, sodium and sulfate in the concentrate and dilute compartments. Here, the concentration of sodium present in EDTA was discounted. Although the initial solution of the synthetic rinsing water was prepared with 0.0014 mol/L of Zn^2+^ (Table 1), or ~90 ppm of Zn^2+^, Table 5 shows that its initial concentration in the experiments, as determined by analytical method, was about 60 ppm. This difference occurred due to the formation of a precipitate with zinc, which was visually observed before the ED tests. The precipitate formation was expected, as shown in the speciation diagram in Figure 4. 

**Figure 8:**
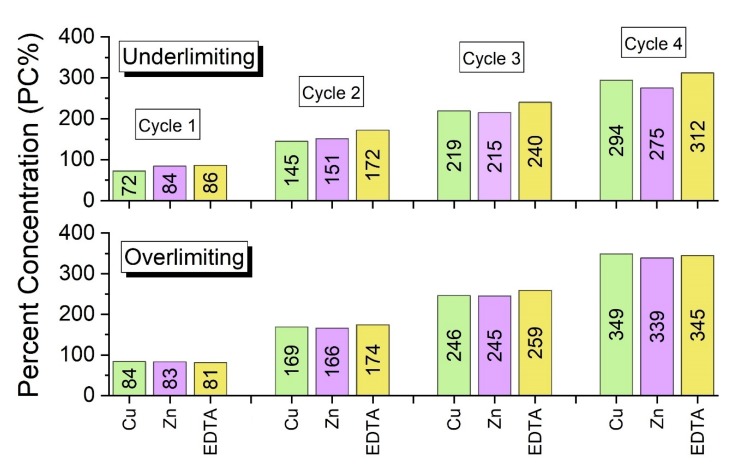
The percent concentrations of Cu, Zn and EDTA obtained in the experiments.

The results of the desalination of the feed solution in each of the four cycles were expected to be similar in the experiments, since the system was carried out in quasi steady-state conditions. The differences shown in Table 5, for both ED tests, are explained by the formation of insoluble species and by their reactions with protons during overlimiting phenomena, as will be discussed. With the data from Table 5, the percent concentrations of Cu, Zn and EDTA were calculated, and this is shown in Figure 8.

According to Figure 8, the species that preferentially crossed the membranes and their concentrations depended on the applied current density, as suggested by Figure 7, regarding the values of the final conductivities of the concentrate compartment. It should be noted that, in general, the highest percent concentrations of copper and zinc were those obtained in the overlimiting test. For EDTA, the percent concentration remained practically constant in the first two cycles, whereas it increased in the third and fourth cycles, in the overlimiting test.

The highest percent concentrations of copper, zinc and EDTA in the overlimiting test can be explained by the occurrence of water dissociation. Zabolotsky et al. [44] studied this phenomenon using different configurations of electrodialysis and observed that in overlimiting conditions, some of the H^+^ ions at the CEM may have migrated from the AEM as a result of water dissociation at this membrane. Hence, the results presented in Figure 8, and those that will be shown, suggest that intense water dissociation occurred on the surface of the AEM in the overlimiting condition. It is well-known that water dissociation occurs mainly at anion-exchange membranes, due to their higher catalytic activity with respect to this phenomenon [45,46]. During the intense migration of hydroxyl ions through the anion-exchange membrane, protons may have accumulated on its cathodic side, leading to a pH decrease. This would have caused the reaction of insoluble species, such as CuO and ZnO, with protons, which would have led to the formation of Cu^2+^ and Zn^2+^ (Equations (3) and (4)). As shown in Figure 4, at a pH lower than approximately 8.3 and 7, there is no ZnO and CuO, respectively, in the working solution. Considering the very dynamic behavior of electrodialysis in relation to the concentration and pH of the diluted solution, especially on the membrane surface, the reactions present in Equations (5) to (8) may also have taken place, which also form Cu^2+^ and Zn^2+^ ions. The values of the equilibrium constant at 25 °C for the equations are from references [47,48], except for Equation (5) and Equation (7). For Equation (5), *log K* was calculated by combining the values from Equation (6) and Equation (9), whereas for Equation (7), *log K* was calculated by combining Equation (8) and Equation (10). The reactions of the hydroxides of copper and zinc with protons are also shown in Equation (11) and Equation (12) [48]. Similar results regarding the decrease in the occurrence of fouling on the AEM-diluate side due to water dissociation were obtained by Cifuentes-Araya et al. [49].

The free metals Cu^2+^ and Zn^2+^ may then have migrated from the AEM to the CEM as a result of the intense electric field and electroconvection, which would have allowed their transport to the concentrate compartment through the cation-exchange membrane. As presented in Table 3, the *i* applied to the overlimiting test was 160% of the *i_lim_* of the CEM, which means that the attraction of cations towards this membrane was very intense. This also explains the current–voltage curve presented in Figure 5 for the CEM, since the resistance of its third region was very low due to the intense transport of cations. The reaction of the complexes of Cu-EDTA and Zn-EDTA with protons also occurred during water dissociation, which led to an increase of EDTA transfer through the anion-exchange membrane, mainly during the third and fourth cycles (Figure 8).
$CuO\left(s\right)+2H^{+}↔Cu^{2+}+H_{2}O$logK=7.66(3)$ZnO\left(s\right)+2H^{+}↔Zn^{2+}+H_{2}O$logK=11.16(4)$Cu{\left(OH\right)}_{2}+H^{+}↔{\left(CuOH\right)}^{+}+H_{2}O$logK=9.30(5)${\left(CuOH\right)}^{+}+H^{+}↔Cu^{2+}+H_{2}O$logK=8.0(6)$Zn{\left(OH\right)}_{2}+H^{+}↔{\left(ZnOH\right)}^{+}+H_{2}O$logK=7.94(7)${\left(ZnOH\right)}^{+}+H^{+}↔Zn^{2+}+H_{2}O$logK=8.96(8)$Cu{\left(OH\right)}_{2}+2H^{+}↔Cu^{2+}+2H_{2}O$logK=17.3(9)$Zn{\left(OH\right)}_{2}+2H^{+}↔Zn^{2+}+2H_{2}O$logK=16.9(10)$Cu{\left(OH\right)}_{2}\left(s\right)+2H^{+}↔Cu^{2+}+2H_{2}O$logK=8.68(11)$Zn{\left(OH\right)}_{2}\left(amorp\right)+2H^{+}↔Zn^{2+}+2H_{2}O$logK=12.48(12)

Finally, it is known that in the case of CEMs, water dissociation is enhanced due to the protonation-deprotonation reactions of metallic precipitates, such as copper and zinc hydroxides and oxides. The general water dissociation reaction involving metal ions was formulated by Ganych et al. [50], and it can be seen in Equations (13) and (14). For AEM, the formation of metal complexes can also catalyze the water dissociation, since they participate as active sites in the protonation-deprotonation reactions [51]. Hence, the presence of insoluble species and metal complexes may also have favored water dissociation at both membranes facing the diluted solution.

(13) $$R-{\left[Metal{\left(H_{2}O\right)}_{2}\right]}^{z+}+H-OH⇄R-{\left[Metal\left(H_{2}O\right)OH\right]}^{\left(z-1\right)+}+H_{3}O^{+}$$

(14) $$R-{\left[Metal\left(H_{2}O\right)OH\right]}^{\left(z-1\right)+}+H-OH⇄R-{\left[Metal{\left(H_{2}O\right)}_{2}\right]}^{z+}+OH^{-}$$

The suggestion of the occurrence of intense water dissociation is also in agreement with the recent work carried out by Barros et al. [23]; in our previous work, we verified, by chronopotentiometry, that an insoluble species was formed at the AEM surface. Oscillations typical of fouling/scaling by insoluble species were observed in the chronopotentiograms, besides the absence of the third region in the current–voltage curve for the solution with the same composition evaluated herein, but with pH = 10.

**Figure 9:**
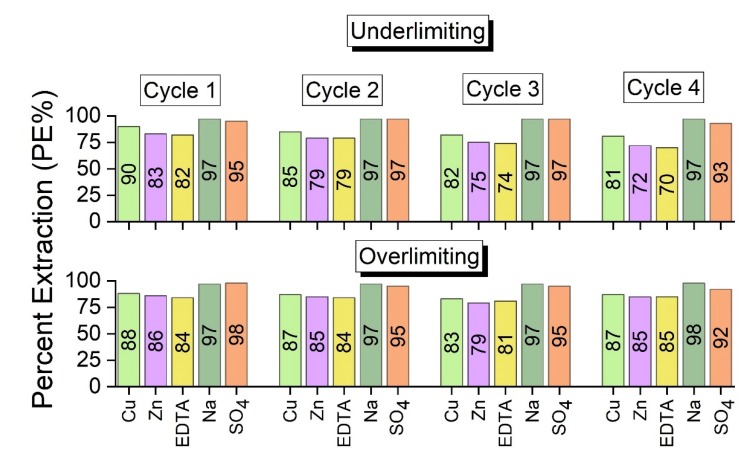
The percent extractions of Cu, Zn, EDTA, Na and SO_4_ obtained in the ED experiments.

With the data from the diluted solutions from Table 5, the percent extractions (PE%) of copper, zinc, EDTA, sodium and sulfate were calculated. As verified in Figure 9, in general, the values of percent extraction of the species are relatively close in both experiments, except for Zn in cycle 2; EDTA in cycle 3; and Cu, Zn and EDTA in cycle 4. 

The percent extraction was calculated as a function of the concentration of the species in the dilute compartment after each cycle in relation to its initial concentration. Therefore, similar PE% values for Cu and Zn were obtained in both experiments because in the underlimiting test, some species were present in the solid state and were not quantified in the chemical analyses, whereas in the overlimiting experiment, these species were transported to the concentrate compartment due to water dissociation. For EDTA, the complex dissociation occurred more intensively during the third and fourth cycles, which explains the highest values of PE%. These results are in accordance with the results for percent concentration already shown and will be confirmed by a mass balance. For Na^+^ and SO_4_^2−^ species, differences in the PE% were not verified by the experiments, since they were already present in the free form (Table 2). Hence, they were not strongly influenced by the water dissociation phenomenon.

#### 3.2.3. Mass Balance

**Figure 10:**
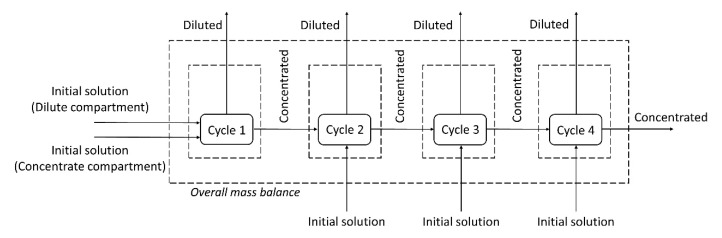
A representation of the mass balance calculated for each cycle and for the overall system.

A mass balance of each species (copper, zinc and EDTA) was performed using the molar flow rates of the species in each cycle. The mass balance for the system was also performed considering all inputs and outputs of the four cycles (overall mass balance), as shown in Figure 10. 

The results of the mass balance are presented in Table 6, which shows the percentage of the species leaving each control volume (diluted and concentrated solutions) in relation to the species entering it. The values slightly above 100% in the mass balance of some species (~3%) are due to the deviations in the chemical analysis. Note that in the underlimiting test, only 91% and 93% of the Cu and Zn, respectively, of the initial solution were present in the final solutions (dilute and concentrate compartments). In turn, the overall mass balance of metals in the overlimiting test was 100%. 

As in the underlimiting experiment, water dissociation (and the reaction of protons with insoluble species) did not occur; part of the metals remained in the solid state and were not quantified in the chemical analysis, which explains why their values were below 100%. These results support our suggestion that the overlimiting experiment led to the reaction of protons with insoluble species of copper and zinc present in the working solution, which allowed the metals passage to the concentrate compartment.

### 3.3. Chronopotentiometric Measurements after the ED Tests

The cation- and anion-exchange membranes were forwarded for chronopotentiometric measurements after both electrodialysis tests, for evaluating their transport properties, such as their limiting current density and ohmic resistance. The virgin membranes, not exposed to electrodialysis, were also evaluated.

**Figure 11:**
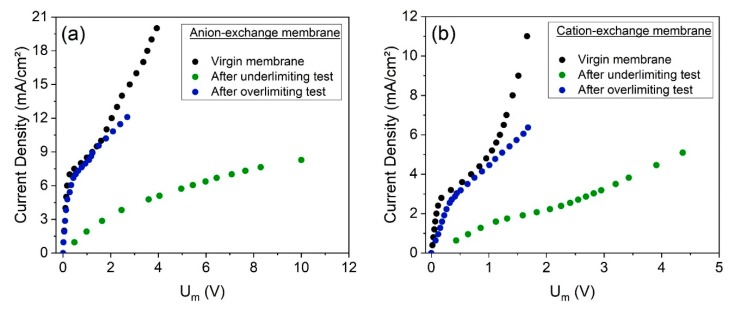
The current–voltage curves obtained by chronopotentiometry of the (**a**) AEM and (**b**) CEM, virgin and after the underlimiting and overlimiting experiments.

Figure 11 presents the current–voltage curves obtained for the AEMs (Figure 11a) and CEMs (Figure 11b) by chronopotentiometry, whereas Table 7 presents the obtained values of limiting current density and ohmic resistance. The errors between the results from the duplicate curves, which are lower than 4%, are also presented in Table 7. 

As observed, the behaviors of the CVCs and the properties obtained for the membranes from the underlimiting test are very distant from those for the virgin membranes. On the other hand, the CVCs of the membranes used in the overlimiting test are very close to those of the virgin membranes. Hence, the overlimiting operation did not cause remarkable modifications in the limiting current density or in the ohmic resistance. 

The CVCs of the membranes used in the overlimiting test suggest the lower tendency of fouling to occur when operating in this condition, which may be explained by the intense electroconvective vortices. Bukhovets et al. [52] proposed the “washing out” effect of electroconvection on organic fouling. According to the authors, the water dissociation phenomenon at the AEM enhances the flux of hydroxyl ions and, together with electroosmotic convection and the effect of current exaltation, contributes to the “washing out” the species fouled. Hence, considering the differences of the current–voltage curves of the CEMs and AEMs after each electrodialysis (Figure 11) and the intense occurrence of water dissociation in the overlimiting test, it may be suggested that in this experiment, fouling/scaling occurrence was not verified in either membrane. Finally, although the “washing out” phenomenon is valid only for anion-exchange membranes, the results here show that the overlimiting operation also helps to mitigate scaling in cation-exchange membranes, as suggested by Mikhaylin et al. [53], but with a lower intensity than for AEMs. 

**Figure 12:**
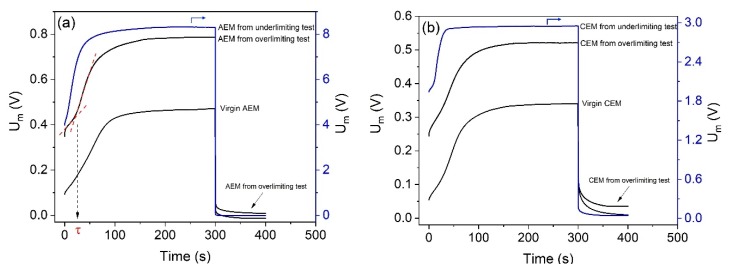
The chronopotentiograms for the (**a**) AEMs under 7.6 mA/cm^2^ and (**b**) CEMs under 3.2 mA/cm^2^. The membranes represented are the virgin ones and those used in electrodialysis in overlimiting and underlimiting conditions.

Figure 12 shows the chronopotentiograms constructed for the anion- (Figure 12a) and cation-exchange membranes (Figure 12b), both after electrodialysis in the overlimiting and underlimiting tests, and virgin. In Figure 12, the current density applied to the AEMs was 7.6 mA/cm^2^, whereas for the CEMs, it was 3.2 mA/cm^2^. The potential drop presented is the total one (measured), in order to show the additional influence of electrodialysis on the ohmic resistance, although in some comparisons of different membranes, some authors represent the “reduced potential drop” by excluding the ohmic potential drop [54]. All curves obtained were typical of monopolar membranes, without the formation of additional inflexion points during the concentration polarization or the relaxation of the system. 

For the anion-exchange membranes (Figure 12a), the initial potential drop of the virgin one was close to 0.1V, whereas for the membranes used in electrodialysis, higher potential drop values were obtained, especially for the AEM from the underlimiting test. This occurred mainly due to the higher ohmic resistance after the electrodialysis [55]. The final values of the potential drop in the steady-state condition also showed very different values after each electrodialysis current mode. Note that for the anion-exchange membrane used in the overlimiting test, the final potential drop is closer to that for the virgin membrane if compared to the AEM from the underlimiting test. This occurred due to the greater membrane resistance after the underlimiting test. For the cation-exchange membranes (Figure 12b), similar behaviors of the AEMs were obtained: for the virgin CEM, the initial potential drop was close to 0.05V, whereas for the membranes used in electrodialysis, higher potential drop values were obtained.

Differences between transition times (τ) were also verified, which correspond to the moment when the concentration of electrolyte at the membrane surface is practically zero and the potential drop tends to infinity. This can be experimentally determined by the intersection of the tangential lines of the first and second stages of the chronopotentiograms [35], as represented in Figure 12a. For *i* = 7.6 mA/cm^2^, the transition time obtained for the virgin AEM was approximately 28 s, whereas for the membranes used in the overlimiting and underlimiting tests, it was 24 s and 6 s, respectively. Hence, the time required for the depletion of counterions in the diffusion boundary layer in the underlimiting test is considerably lower, which means the concentration polarization occurs earlier. This may be explained by the fouling occurrence and the reduction of the fraction of conductive area in the underlimiting test, as verified in the CVC evaluation. For the cation-exchange membranes, transition times also showed remarkable differences between the experiments. For *i* = 3.2 mA/cm^2^, the transition time obtained for the virgin CEM was 29 s, whereas for the membrane after the overlimiting and underlimiting tests, it was 27 s and 11 s, respectively. As verified for the anion-exchange membrane, lower τ values for the CEM after the underlimiting test are due to the occurrence of fouling/scaling. In Figure 12, the potential drop values during the relaxation of the system, i.e., when the current was switched off, showed the following order: membranes from the underlimiting test > membranes from the overlimiting test > virgin membranes. The highest potential drop values for the membranes from the underlimiting test are also due to the presence of fouled species.

The relationship between transition time (τ) and fouling/scaling may also be evaluated by using the modified Sand’s equation [56] (Equation (15)), where ε is the fraction of conductive area, *D* is the electrolyte diffusion coefficient, *C_0_* is the electrolyte concentration at *t* = 0 s, *z* is the counterion charge, *F* is the Faraday constant, *i* is the applied current density, and $\bar{t_{j}}$ and $t_{j}$ are the counterion transport numbers in the membrane and in the solution, respectively. This equation shows that the $i\tau^{1/2}$ values are constant, independent of current density, at a given concentration of electrolytes, and it allows the determination of the fraction of conductive area.

(15) $$i\tau^{1/2}=\frac{\varepsilon C_{0}z_{j}F{\left(\pi D\right)}^{1/2}}{2\left(\bar{t_{j}}-t_{j}\right)}$$

**Figure 13:**
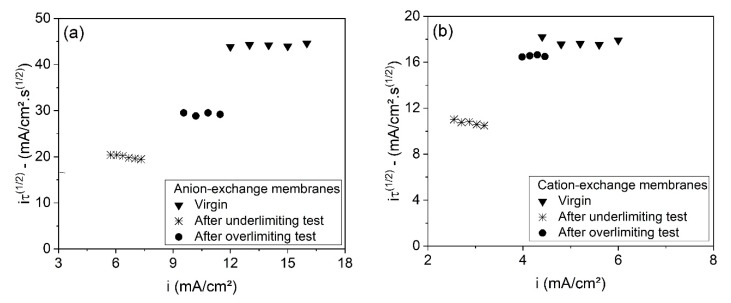
The dependence of $i\tau^{1/2}$ on current density for the (**a**) anion-exchange membranes and (**b**) cation-exchange membranes after the underlimiting and overlimiting tests, as well as for the virgin membranes.

Figure 13 shows the dependence of transition time on current density, represented in Sand’s coordinates for each membrane used in the electrodialysis tests, as well as for the virgin membranes. Here, the plotted transition times were those associated to current densities at least 1.5 times higher than the limiting current density of the membrane/electrolyte systems, as suggested by Mareev et al. [57]. As can be seen, the $i\tau^{1/2}$ values of the anion- (Figure 13a) and cation-exchange membranes (Figure 13b) were fairly constant, independent of the current density, and showed the following order: virgin membranes > membranes from the overlimiting test > membranes from the underlimiting test. This supports the previous discussion about the fraction of conductive area; membranes from the underlimiting test present the lowest ε values due to the occurrence of fouling/scaling. These results confirm the lower tendency of fouling to occur at both membranes when operating in the overlimiting condition. Finally, the greater heterogeneity (lower ε) of the membrane after the underlimiting test may also have favored the “funnel effect” [58], which occurs due to the accumulation of current lines within the well conducting areas of the membrane surface. This also leads to the reduction of transition time, since the potential drop increases more rapidly [59].

## 4. Conclusions

The treatment of wastewater from the brass electroplating industry was evaluated by two electrodialysis tests: one in the underlimiting condition and the other in the overlimiting one, for both membranes. The results suggested the occurrence of intense water dissociation on the cathodic side of the AEM. Although this phenomenon is undesirable in electrodialysis, herein, its occurrence accounted for the highest percent concentrations obtained for copper, zinc and EDTA in the overlimiting test. The water dissociation phenomenon, the reaction of protons with complexes and insoluble species, the intense electric field and the electroconvection may have allowed the migration of the co-ions Cu^2+^ and Zn^2+^ from the AEM to the CEM, favoring their extraction. The improvements in the overlimiting test were obtained due to the electrodialysis current mode used herein, since the concentrate compartments of the cation- and anion-exchange membranes were connected to the same reservoir.

After the electrodialysis, chronopotentiometric tests performed for the CEMs and AEMs showed that the overlimiting operation did not cause remarkable modifications of the limiting current density or ohmic resistance, differently from in the underlimiting test, since electroconvective vortices minimized fouling and scaling at both membranes. This was also verified by differences in the transition times, fractions of conductive area and potential drop values of the chronopotentiograms obtained after each electrodialysis. 

Considering the species that remained in the diluted solutions, the passage of cations through the CEM, and the lower fouling/scaling at the membranes, electrodialysis in the overlimiting condition seems to be more advantageous than that in the underlimiting one. Small intermembrane distances are recommended for the system evaluated in this work, since we verified that the intermembrane distance plays an important role in ionic transfer when water dissociation is dominant. Finally, our work shows that this electrodialysis system has a very promising applicability, particularly to treating solutions with complexes and insoluble species, exploiting the phenomenon of water dissociation.

## Figures and Tables

**Table 1:** The initial conditions of the working solutions.

Molar Concentration					
CuSO_4_.5H_2_O	ZnSO_4_.7H_2_O	EDTA	NaOH	pH	Conductivity (mS/cm)
0.0006	0.0014	0.0015	0.03	12.25	5.3

**Table 2:** The molar concentrations of the main ionic species in the initial composition of the working solution.

	Concentration (mmol/L)
Na^+^	29.44
OH^−^	17.42
SO_4_^2−^	1.75
Zn(EDTA)^2−^	0.83
Zn(EDTA)OH^3−^	0.43
NaSO_4_^−^	0.26
Cu(EDTA)OH^3−^	0.14

**Table 3:** The relationship between the current densities applied to each experiment and the limiting current densities of the membranes.

		Relationship between the *i* Applied and the *i_lim_* of both Membranes	
Experiment	Applied Current Density (mA/cm^2^)	CEM	AEM
Underlimiting	1.2	80% of *i_lim_*	60% of *i_lim_*
Overlimiting	2.4	160% of *i_lim_*	120% of *i_lim_*

**Table 4:** The final pH of the diluted and concentrated solutions after the four cycles of the electrodialysis tests.

	Underlimiting Test		Overlimiting Test	
Cycle	Diluted	Concentrated	Diluted	Concentrated
1st	10.8	12.4	10.7	12.4
2nd	10.2	12.3	10.8	12.5
3rd	10.1	12.3	10.9	12.6
4th	9.9	12.2	10.4	12.6

**Table 5:** The concentrations (in ppm) of copper, zinc, EDTA, sodium and sulfate in the concentrate and dilute compartments.

		Underlimiting Test		Overlimiting Test	
Initial solution	Copper	36		36	
	Zinc	62		59	
	EDTA	544		546	
	Sodium	784		768	
	Sulfate	273		265	
*Electrodialysis*					
		**Underlimiting test**		**Overlimiting test**	
		Diluted	Concentrated	Diluted	Concentrated
Cycle 1	Copper	4	63	4	66
	Zinc	11	114	8	107
	EDTA	100	1013	88	986
	Sodium	25	2322	24	2607
	Sulfate	14	1569	5	2244
Cycle 2	Copper	6	89	5	97
	Zinc	13	156	9	156
	EDTA	117	1478	88	1496
	Sodium	27	3837	18	4284
	Sulfate	9	3457	14	4351
Cycle 3	Copper	7	116	6	124
	Zinc	16	196	12	202
	EDTA	144	1849	103	1962
	Sodium	24	5575	20	5733
	Sulfate	9	5279	13	6375
Cycle 4	Copper	7	143	5	161
	Zinc	18	233	9	257
	EDTA	162	2242	82	2430
	Sodium	20	7477	13	8405
	Sulfate	20	7142	22	9580

**Table 6:** The mass balance of metals and EDTA for each cycle and for the overall system of electrodialysis.

Mass Balance for the Underlimiting Test (%)			
Cycle	Cu	Zn	EDTA
1st	91	100	102
2nd	95	96	102
3rd	98	97	99
4th	99	97	100
Overall system	91	93	102
Mass Balance for the Overlimiting Test (%)			
Cycle	Cu	Zn	EDTA
1st	98	98	98
2nd	100	99	103
3rd	98	100	101
4th	103	102	100
Overall system	101	101	102

**Table 7:** The limiting current density and ohmic resistance of both membranes, virgin and after electrodialysis.

	Anion-Exchange Membrane				Cation-Exchange Membrane			
	*i_lim_* (mA/cm^2^)	Error (%)	Ohmic Resistance (Ω.cm^2^)	Error (%)	*i_lim_* (mA/cm^2^)	Error (%)	Ohmic Resistance (Ω.cm^2^)	Error (%)
Virgin	6.9	2.2	28	0.3	2.7	2.1	44	3.4
After ED (underlimiting)	3.7	0.9	572	2.0	1.6	0.2	672	1.9
After ED (overlimiting)	6.3	0.4	31	3.2	2.6	1.7	118	3.9

## References

[1] Rashwan S.M. (2007). Electrodeposition of Zn–Cu coatings from alkaline sulphate bath containing glycine. Trans. Inst. Met. Finish..

[2] Ramírez C., Calderón J. (2016). Study of the effect of Triethanolamine as a chelating agent in the simultaneous electrodeposition of copper and zinc from non-cyanide electrolytes. J. Electroanal. Chem..

[3] Ballesteros J., Torres-Martínez L.M., Juárez-Ramírez I., Trejo G., Meas-Vong Y. (2014). Study of the electrochemical co-reduction of Cu^2+^ and Zn^2+^ ions from an alkaline non-cyanide solution containing glycine. J. Electroanal. Chem..

[4] Vagramyan T., Leach J., Moon J. (1979). On the problems of electrodepositing brass from non-cyanide electrolytes. Electrochim. Acta.

[5] Brenner A. (1963). Electrodeposition of Alloys.

[6] Carlos I., De Almeida M.R.H. (2004). Study of the influence of the polyalcohol sorbitol on the electrodeposition of copper–zinc films from a non-cyanide bath. J. Electroanal. Chem..

[7] De Almeida M., Barbano E., De Carvalho M., Tulio P., Carlos I. (2015). Copper–zinc electrodeposition in alkaline-sorbitol medium: Electrochemical studies and structural, morphological and chemical composition characterization. Appl. Surf. Sci..

[8] De Almeida M.R.H., Barbano E.P., Zacarin M.G., De Brito M.M., Tulio P.C., Carlos I.A. (2016). Electrodeposition of CuZn films from free-of-cyanide alkaline baths containing EDTA as complexing agent. Surf. Coat. Technol..

[9] De Almeida M., Barbano E., De Carvalho M., Carlos I., Siqueira J., Barbosa L. (2011). Electrodeposition of copper–zinc from an alkaline bath based on EDTA. Surf. Coat. Technol..

[10] Assaf F.H., Rehim S.S.A.E., Mohamed A.S., Zaky A.M. (1995). Electroplating of brass from citrate-based alloy baths. Indian J. Chem..

[11] De Senna L.F., Díaz S., Sathler L. (2003). Electrodeposition of copper–zinc alloys in pyrophosphate-based electrolytes. J. Appl. Electrochem..

[12] Despic A., Marinovic V., Jović V. (1992). Kinetics of deposition and dissolution of brass from the pyrophosphate—Oxalate bath. J. Electroanal. Chem..

[13] Fujiwara Y., Enomoto H. (1988). Characterization of Cu-Zn alloy deposits from glucoheptonate baths. Surf. Coat. Technol..

[14] Krishnan R., Muralidharan V., Natarajan S. (1996). A non-cyanide brass plating bath. Bull. Electrochem..

[15] De Filippo D., Rossi A., Atzei D. (1992). A tartrate-based alloy bath for brass-plated steel wire production. J. Appl. Electrochem..

[16] De Vreese P., Skoczylas A., Matthijs E., Fransaer J., Binnemans K. (2013). Electrodeposition of copper–zinc alloys from an ionic liquid-like choline acetate electrolyte. Electrochim. Acta.

[17] Rousse C., Beaufils S., Fricoteaux P. (2013). Electrodeposition of Cu–Zn thin films from room temperature ionic liquid. Electrochim. Acta.

[18] Juskenas R., Karpavičienė V., Pakštas V., Selskis A., Kapočius V. (2007). Electrochemical and XRD studies of Cu–Zn coatings electrodeposited in solution with d-mannitol. J. Electroanal. Chem..

[19] Barbano E., De Oliveira G., De Carvalho M., Carlos I. (2014). Copper–tin electrodeposition from an acid solution containing EDTA added. Surf. Coat. Technol..

[20] De Oliveira G., Carlos I. (2009). Silver–zinc electrodeposition from a thiourea solution with added EDTA or HEDTA. Electrochim. Acta.

[21] Cherif A., Elmidaoui A., Gavach C. (1993). Separation of Ag^+^, Zn^2+^ and Cu^2+^ ions by electrodialysis with monovalent cation specific membrane and EDTA. J. Membr. Sci..

[22] Iizuka A., Yamashita Y., Nagasawa H., Yamasaki A., Yanagisawa Y. (2013). Separation of lithium and cobalt from waste lithium-ion batteries via bipolar membrane electrodialysis coupled with chelation. Sep. Purif. Technol..

[23] Barros K.S., Espinosa D. (2018). Chronopotentiometry of an anion-exchange membrane for treating a synthesized free-cyanide effluent from brass electrodeposition with EDTA as chelating agent. Sep. Purif. Technol..

[24] Benvenuti T., Rodrigues M.A.S., Bernardes A.M., Ferreira J.Z. (2017). Closing the loop in the electroplating industry by electrodialysis. J. Clean. Prod..

[25] Marder L., Bernardes A.M., Ferreira J.Z. (2004). Cadmium electroplating wastewater treatment using a laboratory-scale electrodialysis system. Sep. Purif. Technol..

[26] Bittencourt S.D., Marder L., Benvenuti T., Ferreira J.Z., Bernardes A.M. (2017). Analysis of different current density conditions in the electrodialysis of zinc electroplating process solution. Sep. Sci. Technol..

[27] Belova E.I., Lopatkova G.Y., Pismenskaya N.D., Nikonenko V., Larchet C., Pourcelly G. (2006). Effect of Anion-exchange Membrane Surface Properties on Mechanisms of Overlimiting Mass Transfer. J. Phys. Chem. B.

[28] Pismenskaya N., Nikonenko V., Zabolotsky V.I., Sandoux R., Pourcelly G., Tskhay A.A. (2008). Effects of the desalination chamber design on the mass-transfer characteristics of electrodialysis apparatuses at overlimiting current densities. Russ. J. Electrochem..

[29] Nikonenko V., Kovalenko A., Urtenov M.K., Pismenskaya N.D., Han J., Sistat P., Pourcelly G. (2014). Desalination at overlimiting currents: State-of-the-art and perspectives. Desalination.

[30] Kniaginicheva E., Pismenskaya N., Melnikov S., Belashova E., Sistat P., Cretin M., Nikonenko V. (2015). Water splitting at an anion-exchange membrane as studied by impedance spectroscopy. J. Membr. Sci..

[31] Lemay N., Mikhaylin S., Bazinet L. (2019). Voltage spike and electroconvective vortices generation during electrodialysis under pulsed electric field: Impact on demineralization process efficiency and energy consumption. Innov. Food Sci. Emerg. Technol..

[32] Lemay N., Mikhaylin S., Mareev S., Pismenskaya N., Nikonenko V., Bazinet L. (2020). How demineralization duration by electrodialysis under high frequency pulsed electric field can be the same as in continuous current condition and that for better performances?. J. Memb. Sci..

[33] Dufton G., Mikhaylin S., Gaaloul S., Bazinet L. (2020). Systematic Study of the Impact of Pulsed Electric Field Parameters (Pulse/Pause Duration and Frequency) on ED Performances during Acid Whey Treatment. Membranes.

[34] Sosa-Fernandez P., Post J., Ramdlan M., Leermakers F., Bruning H., Rijnaarts H. (2020). Improving the performance of polymer-flooding produced water electrodialysis through the application of pulsed electric field. Desalination.

[35] Barros K.S., Scarazzato T., Espinosa D. (2018). Evaluation of the effect of the solution concentration and membrane morphology on the transport properties of Cu(II) through two monopolar cation–exchange membranes. Sep. Purif. Technol..

[36] Benvenuti T., Krapf R., Rodrigues M.A.S., Bernardes A.M., Ferreira J.Z. (2014). Recovery of nickel and water from nickel electroplating wastewater by electrodialysis. Sep. Purif. Technol..

[37] Scarazzato T., Panossian Z., Tenório J., Pérez-Herranz V., Espinosa D. (2018). Water reclamation and chemicals recovery from a novel cyanide-free copper plating bath using electrodialysis membrane process. Desalination.

[38] Buzzi D., Viegas L.S., Rodrigues M.A.S., Bernardes A.M., Tenório J. (2013). Water recovery from acid mine drainage by electrodialysis. Miner. Eng..

[39] Scarazzato T., Panossian Z., García-Gabaldón M., Ortega E., Tenório J., Pérez-Herranz V., Espinosa D. (2017). Evaluation of the transport properties of copper ions through a heterogeneous ion-exchange membrane in etidronic acid solutions by chronopotentiometry. J. Membr. Sci..

[40] Puigdomench I. (2001). Hydra Medusa—Make Equilibrium Diagrams Using Sophisticated Algorithms.

[41] Melnikova E., Pismenskaya N., Bazinet L., Mikhaylin S., Nikonenko V. (2018). Effect of ampholyte nature on current-voltage characteristic of anion-exchange membrane. Electrochim. Acta.

[42] Martí-Calatayud M., García-Gabaldón M., Pérez-Herranz V. (2013). Effect of the equilibria of multivalent metal sulfates on the transport through cation-exchange membranes at different current regimes. J. Membr. Sci..

[43] Pismenskaya N., Nikonenko V., Auclair B., Pourcelly G. (2001). Transport of weak-electrolyte anions through anion exchange membranes—Current-voltage characteristics. J. Memb. Sci..

[44] Zabolotsky V., Nikonenko V., Pismenskaya N., Laktionov E., Urtenov M., Strathmann H., Wessling M., Koops G. (1998). Coupled transport phenomena in overlimiting current electrodialysis. Sep. Purif. Technol..

[45] Król J. (1999). Concentration polarization with monopolar ion exchange membranes: Current-voltage curves and water dissociation. J. Membr. Sci..

[46] Belloň T., Polezhaev P., Vobecká L., Svoboda M., Slouka Z. (2019). Experimental observation of phenomena developing on ion-exchange systems during current-voltage curve measurement. J. Membr. Sci..

[47] Baes C.F., Mesmer R.E. (1976). The Hydrolysis of Cations.

[48] Lindsay W.L. (1979). Chemical Equilibria in Soils.

[49] Cifuentes-Araya N., Astudillo-Castro C., Bazinet L. (2014). Mechanisms of mineral membrane fouling growth modulated by pulsed modes of current during electrodialysis: Evidences of water splitting implications in the appearance of the amorphous phases of magnesium hydroxide and calcium carbonate. J. Colloid Interface Sci..

[50] Ganych V.V., Zabolotskii V.I., Shel’deshov N.V. (1992). Electrolytic dissociation of water molecules in systems comprising solutions and MA-40 anion-exchange membranes modified with transition metal ions. Sov. Electrochem..

[51] Zabolotskii V.I., Ganych V.V., Sheldeshov N.V. (1991). Effect of Copper Complexes with the Ionogenic Groups of the MA-40 Anion-Exchange Membrane on the Dissociation Rate of Water Molecules in the Electric Field. Sov. Electrochem..

[52] Bukhovets A., Eliseeva T., Dalthrope N., Oren Y. (2011). The influence of current density on the electrochemical properties of anion-exchange membranes in electrodialysis of phenylalanine solution. Electrochim. Acta.

[53] Mikhaylin S., Nikonenko V., Pismenskaya N., Pourcelly G., Choi S., Kwon H.J., Han J., Bazinet L. (2016). How physico-chemical and surface properties of cation-exchange membrane affect membrane scaling and electroconvective vortices: Influence on performance of electrodialysis with pulsed electric field. Desalination.

[54] Gil V., Andreeva M., Jansezian L., Han J., Pismenskaya N., Nikonenko V., Larchet C., Dammak L. (2018). Impact of heterogeneous cation-exchange membrane surface modification on chronopotentiometric and current–voltage characteristics in NaCl, CaCl2 and MgCl2 solutions. Electrochim. Acta.

[55] Korzhova E., Pismenskaya N., Lopatin D., Baranov O., Dammak L., Nikonenko V. (2016). Effect of surface hydrophobization on chronopotentiometric behavior of an AMX anion-exchange membrane at overlimiting currents. J. Membr. Sci..

[56] Choi J. (2001). Pore size characterization of cation-exchange membranes by chronopotentiometry using homologous amine ions. J. Membr. Sci..

[57] Mareev S., Butylskii D., Pismenskaya N., Nikonenko V. (2016). Chronopotentiometry of ion-exchange membranes in the overlimiting current range. Transition time for a finite-length diffusion layer: Modeling and experiment. J. Membr. Sci..

[58] Rubinstein I., Zaltzman B., Pundik T. (2002). Ion-exchange funneling in thin-film coating modification of heterogeneous electrodialysis membranes. Phys. Rev. E.

[59] Andreeva M., Gil V., Pismenskaya N., Nikonenko V., Dammak L., Larchet C., Grande D., Kononenko N. (2017). Effect of homogenization and hydrophobization of a cation-exchange membrane surface on its scaling in the presence of calcium and magnesium chlorides during electrodialysis. J. Membr. Sci..

